# Structure-Function Analysis of Rgs1 in *Magnaporthe oryzae*: Role of DEP Domains in Subcellular Targeting

**DOI:** 10.1371/journal.pone.0041084

**Published:** 2012-07-19

**Authors:** Ravikrishna Ramanujam, Xu Yishi, Hao Liu, Naweed I. Naqvi

**Affiliations:** 1 Fungal Patho-Biology Group, Temasek Life Sciences Laboratory, Singapore, Singapore; 2 School of Biological Sciences, Nanyang Technological University, Singapore, Singapore; 3 MOE Key Laboratory of Industrial Fermentation Microbiology, College of Biotechnology, Tianjin University of Science and Technology, Tianjin, China; 4 Department of Biological Sciences, National University of Singapore, Singapore, Singapore; Seoul National University, Republic of Korea

## Abstract

**Background:**

Rgs1, a prototypical Regulator of G protein Signaling, negatively modulates the cyclic AMP pathway thereby influencing various aspects of asexual development and pathogenesis in the rice-blast fungus *Magnaporthe oryzae*. Rgs1 possesses tandem DEP motifs (termed DEP-A and DEP-B; for *D*ishevelled, *E*gl-10, *P*leckstrin) at the N-terminus, and a Gα-GTP interacting RGS catalytic core domain at the C-terminus. In this study, we focused on gaining further insights into the mechanisms of Rgs1 regulation and subcellular localization by characterizing the role(s) of the individual domains and the full-length protein during asexual development and pathogenesis in *Magnaporthe*.

**Methodology/Principal Findings:**

Utilizing western blot analysis and specific antisera against the N- and C-terminal halves of Rgs1, we identify and report the *in vivo* endoproteolytic processing/cleavage of full-length Rgs1 that yields an N-terminal DEP and a RGS core domain. Independent expression of the resultant DEP-DEP half (N-Rgs1) or RGS core (C-Rgs1) fragments, failed to complement the *rgs1*Δ defects in colony morphology, aerial hyphal growth, surface hydrophobicity, conidiation, appressorium formation and infection. Interestingly, the full-length Rgs1-mCherry, as well as the tagged N-terminal DEP domains (individually or in conjunction) localized to distinct punctate vesicular structures in the cytosol, while the catalytic RGS core motif was predominantly vacuolar.

**Conclusions/Significance:**

Based on our data from sequence alignments, immuno-blot and microscopic analysis, we propose that the post-translational proteolytic processing of Rgs1 and the vacuolar sequestration of the catalytic RGS domain represents an important means of down regulating Rgs1 function and thus forming an additional and alternative means of regulating G protein signaling in *Magnaporthe*. We further hypothesize the prevalence of analogous mechanisms functioning in other filamentous fungi. Furthermore, we conclusively assign a specific vesicular/membrane targeting function for the N-terminal DEP domains of Rgs1 in the rice-blast fungus.

## Introduction

In several eukaryotes, including pathogenic fungi, heterotrimeric (αβγ) guanine-nucleotide binding proteins (G proteins) function to mediate the transfer of external environmental stimuli to downstream intracellular signaling components, which in turn regulate several important aspects of growth, development and morphogenesis. In *Magnaporthe oryzae*, G protein mediated cAMP signaling is crucial for regulating various aspects of growth, conidiation, appressorium formation and function [1], [2], [3], [4], [5], [6]. G protein signaling is initiated upon ligand binding to upstream seven transmembrane receptors known as G protein-coupled receptors (GPCRs). Upon ligand binding, the GPCR undergoes a conformational change causing the receptor to function as a guanine nucleotide exchange factor (GEF) promoting exchange of GDP to GTP on the Gα subunit. Such nucleotide exchange leads to the dissociation of Gα from the Gβγ heterodimer [7]. Both Gα-GTP and Gβγ then activate various downstream effectors, which include adenylyl cyclase, phospholipases, ion channels and phosphodiesterases [8]. Active signaling lasts until the Gα subunit hydrolyzes the bound GTP to GDP by its intrinsic GTPase activity, allowing Gα to re-associate with Gβγ to form an inactive complex and thus initiating a new cycle of signaling. Therefore, the duration of active signaling by the G protein is dependent on the guanine nucleotide state of Gα subunit [6], [7]. RGS proteins function by stabilizing the “switch” regions on the Gα subunits, which undergo conformational change upon GTP hydrolysis. Stabilization of the transition state confirmation is believed to lower the energy of activation, thereby leading to an increase (10–1000 folds) in the rate of the reaction [9], [10], [11], [12], [13].

Proper control of the specificity and intensity of G protein signaling is vital for the accurate translation of signals into appropriate and precise cellular responses. RGS proteins, act towards accelerating the hydrolysis of GTP to GDP on the active Gα subunits [14], [15]. Thus, RGSs constitute a crucial element regulating the intensity and duration of G protein signaling.

RGS proteins are evolutionarily conserved in yeasts like *S. cerevisiae*, *S. pombe* [16], [17] and also in many filamentous fungi such as *Aspergillus, Candida, Cryptococcus, Cryphonectria, Ustilago, Fusarium, Magnaporthe* and *Metarhizium* [2], [18], [19], [20], [21], [22], [23], [24], [25]. Work carried out on the *Saccharomyces* RGS protein (Sst2) has shown that disruption of Sst2 function increases pheromone sensitivity by 100 to 300 fold, and furthermore the cells permanently arrest in the G1 phase of the cell cycle [26]. Conversely, overexpression of Sst2 or gain-of-function mutations in Sst2 dramatically dampens pheromone induced cell cycle arrest [26], [27]. Previous work carried out in our lab on the Sst2 ortholog Rgs1 (MGG_14517) in the filamentous phytopathogen *Magnaporthe oryzae*, describes a conserved function for Rgs1 as a negative regulator of G protein signaling, in addition to facilitating responses towards host thigmotropic or hardness cues [2]. We also found that the *rgs1*Δ strain was compromised in aspects of conidiation, wettability, inductive surface signaling, maintenance of intracellular cAMP levels and pathogenicity [2], [28]. More recently, an elegant study by Zhang.H *et al*., characterized seven additional members of the RGS family. The authors have further implicated Rgs1 in regulating cell wall integrity, surface hydrophobicity, sexual development and *in-planta* growth [29].

Most fungal RGS proteins have two DEP domains in tandem (a degenerate DEP-A and a conserved DEP-B, nearly 80 aa long) at the N-terminal portion of the protein and a highly conserved RGS catalytic core domain at the C- terminus (∼120 aa) [20], [28], [30]. The existence of repeated DEP domains in Rgs is supposedly unique to fungi and this domain has been implicated in membrane targeting of RGS proteins to the Golgi and plasma membrane [31], [32]. Although a number of *in vitro* studies have clearly demonstrated that the catalytic RGS domain as necessary and sufficient for the GAP activity exhibited by the RGS/Sst2 protein, *in vivo* studies have shown that even modest truncations at the N-terminal portion, completely abolishes RGS/Sst2 protein function [26], [33]. Thus, an intact DEP-DEP domain plays a crucial role in the proper function of the RGS protein.

Hoffman *et al*., have previously demonstrated that yeast Sst2 undergoes an endoproteolytic cleavage releasing separate but functional N-terminal DEP-DEP and C-terminal RGS core/catalytic domain fragments *in vivo* [34]. In this study, we assess whether *Magnaporthe* Rgs1 also undergoes endoproteolytic cleavage. Secondly, we were also interested in deciphering the functional importance, if any, of such cleavage products, and finally to follow their intracellular fate/subcellular localization. Towards this end, we employed immuno-blot as well as imaging techniques. Based on previously published experimental evidence on the yeast Sst2, we generated *Magnaporthe* strains specifically expressing mCherry-tagged versions of the DEP-DEP domains (either DEP-A and DEP-B together or individually) or only the RGS catalytic core domain in the *rgs1*Δ background. In addition, we generated a C-terminal tagged version of the full length Rgs1. We further compared and characterized the domain deletion strains in relation to the *rgs1*Δ defects. Using wide-field fluorescence microscopy we further assessed the subcellular localization of Rgs1 and the contributions(s) of the individual domains or the full-length protein.

## Results

### Domain Organization and Sequence Alignment of Rgs1

**Figure 1:**
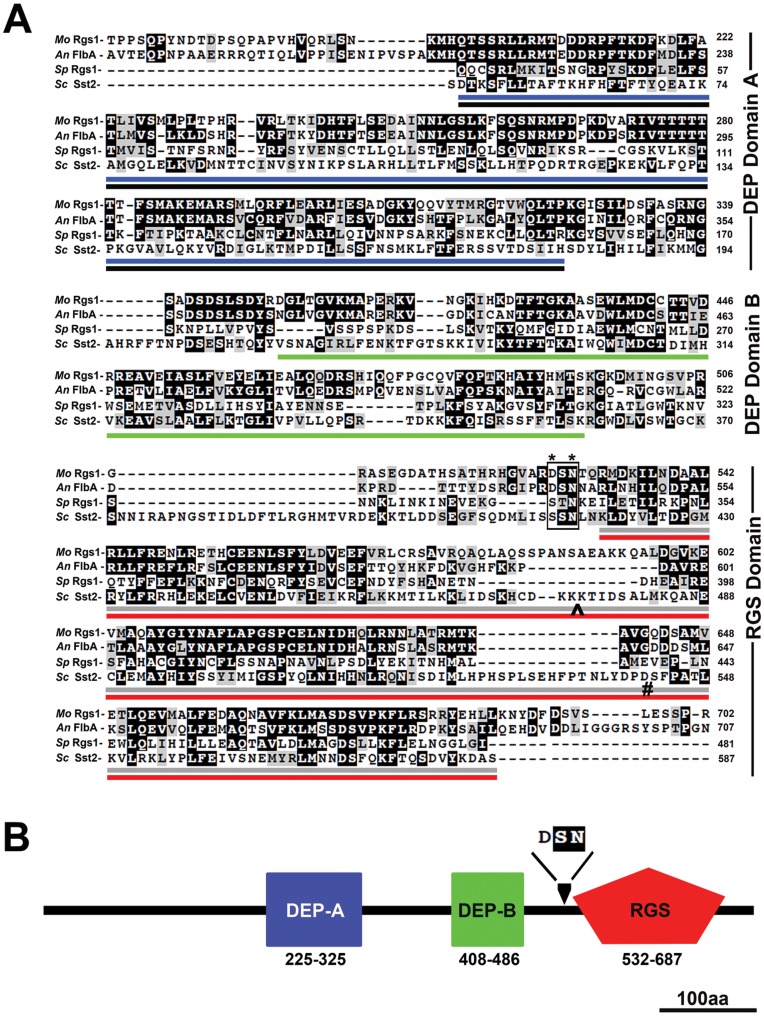
Comparative sequence alignment of other fungal RGS proteins and organization of conserved domains in *Magnaporthe* Rgs1. (**A**) Sequence alignment of RGS proteins from *M. oryzae* (EAH54653/MGG_01417), *A. nidulans* (P38093), *S. pombe* (Q09777) and *S. cerevisiae* (AAA35104). The double DEP domains as well as the core RGS domains are conserved in filamentous and yeast species. The position of the DEP-A domain is underlined in blue/black. Similarly, the shorter DEP-B domain is highlighted in green and the catalytic RGS core domain in red/gray. The asterisks indicate the putative cleavage sites (S-Serine and N-Asparagine), as identified for *S. cerevisiae* Sst2. The RGS domain of Sst2 was truncated at positions # and ^ for purposes of alignment. The conserved residues are shaded in black while similar residues are in gray. (**B**) The N terminal region contains a non-canonical DEP-A domain (225–325aa) and a second canonical DEP-B domain (408–486aa). The catalytic RGS core domain (532–687aa) resides at the C-terminal end of the protein. The arrowhead indicates the region where endoproteolytic cleavage likely occurs.

*Magnaporthe* Rgs1 contains two DEP domains at the N terminal portion of the protein; the first being a prototypical or canonical DEP domain (408–486aa), which is preceded by an atypical DEP-like motif between aa 225–325. At the C terminus is a highly conserved Gα-GTP interacting RGS catalytic core (532–687aa) (Figure 1A and 1B). Between the double DEP domain and the RGS domain is a short stretch of 37 amino acids, within which resides a putative endoproteolytic cleavage site. In the yeast Sst2, the serine (S) at position 415 and asparagine (N) at position 417 have been identified as the likely sites of proteolytic cleavage [34]. Based on our sequence alignment, we found that the asparagine (N) is conserved in filamentous fungi. However, the serine (S, a polar uncharged aa) is replaced by aspartic acid (D, a polar acidic aa) only in the filamentous counterparts (Figure 1A, highlighted by box and asterisks).

Thus, based on data obtained from domain and sequence alignment analysis, we infer that *Magnaporthe* Rgs1, in addition to being functionally similar to *S. cerevisiae* Sst2 [2], is also architecturally identical, and likely retains a potentially conserved endoproteolytic cleavage site between the N and C terminal halves.

### Endoproteolytic Cleavage of *Magnaporthe* Rgs1

**Figure 2:**
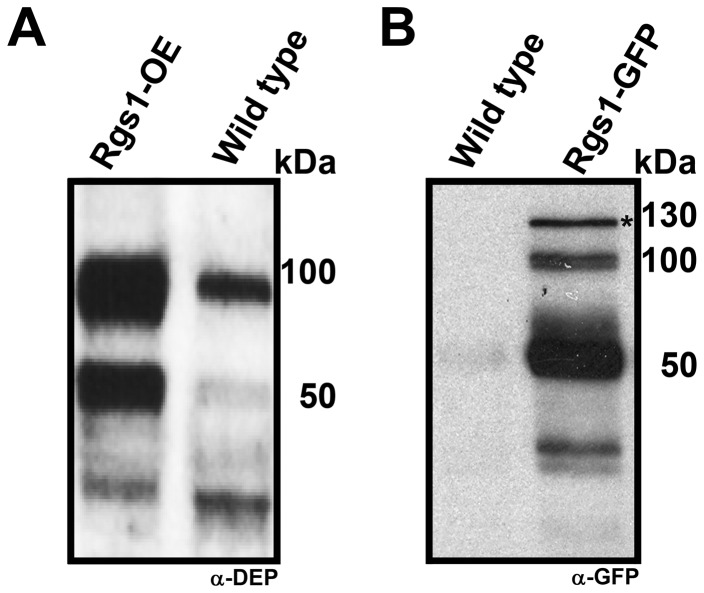
Confirmation of endoproteolytic cleavage of Rgs1 in *Magnaporthe.* Rgs1 in *Magnaporthe* undergoes endoproteolytic cleavage. (**A** and **B**) Total protein extracts were prepared from the Rgs1 over expression (Rgs1-OE) or Rgs1-GFP strains. The lysates were resolved by SDS-PAGE and analyzed by western blot using antibodies against DEP-DEP domain (α-DEP) and GFP (α-GFP). Specific immuno reactive bands corresponding to the N- and C- terminal endoproteolytic cleavage products of Rgs1 were detected in each case and are described in detail in the results section. The wild type served as a control. Asterisk indicates a probable post-translationally modified form of Rgs1. Molecular mass standards in kDa are indicted on the right.

In order to assess whether *Magnaporthe* Rgs1 undergoes endoproteolytic cleavage *in vivo*, we carried out western blot analysis of whole cell extracts from the wild-type Rgs1-OE and Rgs1-GFP strains. The Rgs1-OE is a previously characterized strain that over produces Rgs1 protein as a result of extra copies of the *RGS1* gene [2], [28]. In the Rgs1-GFP strain, Rgs1 is C-terminally tagged with eGFP under native regulation. To specifically detect the cleaved N-terminal DEP fragment, we performed immuno-blot analysis on whole cell lysates from the Rgs1-OE strain with a polyclonal antibody specifically raised against the N terminal DEP-DEP domain [2], [28] (α-DEP antibody). As shown in Figure 2A, the full-length protein (∼80 kDa, Rgs1) and the N- terminal cleaved fragment of ∼55 kDa (DEP-DEP domain, as confirmed by mass spectrometry) were observed, supporting the possibility that *Magnaporthe* Rgs1 undergoes endoproteolytic cleavage at the predicted site akin to Sst2. A similar cleavage pattern was also observed for Rgs1 in the wild type, further substantiating such post-translational processing (Figure 2A, lane 2). Next, in order to confirm the cleaved RGS catalytic core, we repeated the western blot analysis on whole cell lysates of Rgs1-GFP strain with anti-GFP antibodies (Figure 2B). Two major bands, representing the full-length Rgs1-GFP (∼110 kDa) and the cleaved RGS domain fused to GFP (∼50 kDa, RGS-GFP) were observed (Figure 2B, lane 2). Total protein extracts from the wild type served as a control (Figure 2B, lane 1).

Taken together, we conclude that, *Magnaporthe* Rgs1 undergoes proteolytic processing, post-translationally, likely at the conserved predicted site (Figure 1B and 2), to yield separate DEP-DEP and RGS domains.

### Colony Morphology and Aerial Hyphal Growth

**Figure 3:**
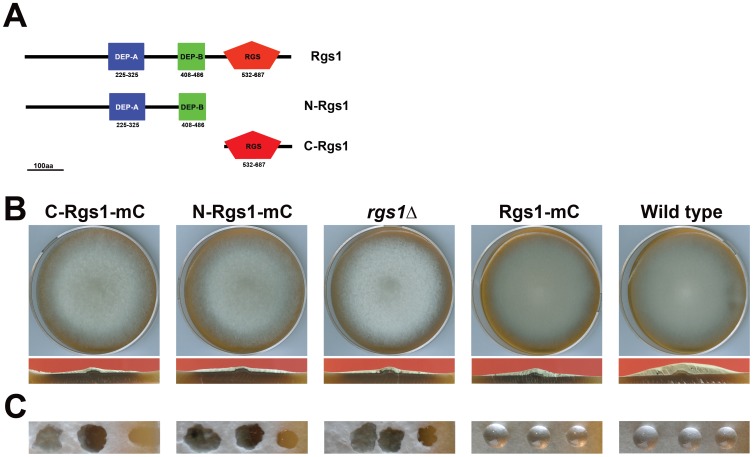
Expression of individual domain fragments fails to rescue colony morphology, aerial hypal growth, surface hydrophobicity and hyperconidiation defects displayed by *rgs1*Δ strain. (A) Schematic depicting the DEP domain A (blue) and domain B (green) and the RGS domain (red) in the full-length protein (Rgs1). The N-Rgs1 includes amino acids 1–225, as well as the tandem DEP domains (225–486aa). The C-Rgs1 includes only the RGS domain (532–714aa). (B) Morphology of the C-Rgs1-mC, N-Rgs1-mC, *rgs1*Δ, Rgs1-mC and wild type colonies. The upper panels are photographs of the indicated strains taken after a week of growth on prune agar medium in the dark. The lower panels depict the cross sections of the above colonies at medial planes. The individual domain expressing strains are identical to the *rgs1*Δ strain. (C) The water soaking ability/phenotype of the C-Rgs1-mC, N-Rgs1-mC, *rgs1*Δ, Rgs1-mCherry and wild-type colonies. The panels show photographs of the surface of the colonies. The C-Rgs1-mC, N-Rgs1-mC expressing strains retain their ability to soak droplets of water inoculated on the colony surface.

In order to characterize the function of the individual domains of Rgs1 in *Magnaporthe*, either the DEP-DEP (termed N-Rgs1) or the RGS domain (C-Rgs1) tagged to fluorescent protein mCherry (mC) were expressed in the *rgs1*Δ background. The transformed strains were grown on Prune Agar (PA) medium for 7 days at 28°C in the dark, and characterized. Expression of the individual domain fragments was also confirmed by western blot analysis. Compared to the wild type, the *rgs1*Δ strain did not show any obvious defects during radial growth, however appeared flat as a result of reduced aerial hyphal development [2], [28] (Figure 3B, upper and lower panels). We found that the strains expressing either the DEP-DEP domain (N-Rgs1-mC) or the RGS domain (C-Rgs1-mC) alone exhibited identical radial growth patterns as the *rgs1*Δ and failed to suppress the flat colony morphology displayed by the *rgs1*Δ (Figure 3B). These results indicate that expression of individual N- or C-terminal domains of Rgs1 is not sufficient to rescue the aerial hyphal growth defects upon loss of Rgs1 function. We concur that both the domains are likely necessary for the function of Rgs1 in proper asexual differentiation.

### Surface Hydrophobicity and Wettability

A characteristic defect displayed by the *rgs1*Δ is the wettability phenotype, the soaking of water droplets inoculated on the surface of the colony upon prolonged incubation (within 15–30 min) [28], [29]. It has been further suggested that the water soaking ability of the colony is related to the mycelial hydrophobicity which may in turn be regulated by the hydrophobin genes *MPG1* and *MHP1* [35], [36]. We tested if the *rgs1*Δ strain expressing the individual domains was still wettable. In contrast to the wild type or the Rgs1-mC expressing strains, in which the inoculated droplets persisted for extended periods, the droplets inoculated on the surface of the *rgs1*Δ, DEP-DEP domain lacking (C-Rgs1) and the RGS domain lacking (N-Rgs1) colonies were rapidly taken up within 30 min post inoculation (Figure 3C). In conclusion, surface hydrophobicity is regulated by G proteins in *Magnaporthe* and likely requires an intact Rgs1 as a key regulator of this process.

### Conidiation in the Domain Deletion Mutants

Rgs1 has been shown previously to be a negative regulator of conidiation and the *rgs1*Δ strain produces nearly three-fold higher number of conidia compared to the wild type [2]. Given that the domain deletion strains were identical to the *rgs1*Δ in terms of colony morphology, aerial hyphal growth and wettability we were interested to determine if the expression of the individual domains could suppress the conidiation defects of *rgs1*Δ.

**Figure 4:**
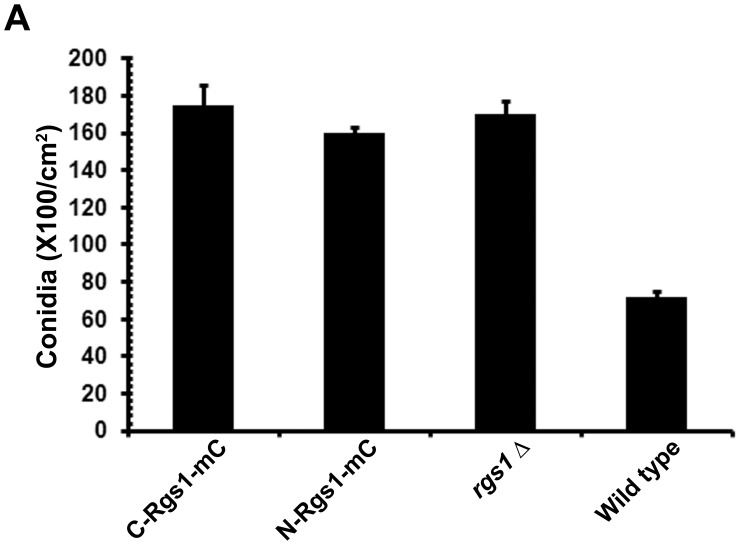
(A) Comparative quantitative analysis of conidiation in the C-Rgs1-mC, N-Rgs1–mC, *rgs1*Δ and wild-type strain. The indicated strains were grown in the dark for a day followed by exposure to constant illumination for a week. Data represents mean ± SE of three independent replicates for the assessment of conidiation in each strain.

Conidia were harvested and quantified from the individual domain deletion, wild-type and *rgs1*Δ strains. As expected, we found that the *rgs1*Δ strain hyper conidiated and produced ∼2.4 fold (p<0.01) higher number of conidia compared to the wild type (Figure 4A). The C-Rgs1 strain (lacking the DEP-DEP) as well as the N-Rgs1-mC strain expressing strain (deleted for the RGS) behaved similar to the *rgs1*Δ, producing ∼2–3 fold (p<0.01 and p<0.05 respectively) higher conidia compared to the wild type.

Taken together, our results suggest that the C-Rgs1 and N-Rgs1 strains display a hyper conidiation defect similar to the *rgs1*Δ strain and expression of the individual domains independently failed to suppress the conidiation defects in the *rgs1*Δ.

### Effect on Appressorium Formation and Efficiency

Surface cues such as hardness and hydrophobicity are known to play an important role in determining the efficiency of appressorium formation. For *in vitro* appressorial assays, artificial membranes can be used to mimic plant surface characteristics. The wild type readily forms mature appressoria on inductive surfaces (e.g. hydrophobic plastic coverslip), but is incapable of doing so on non-inductive surfaces (hydrophilic GelBond membrane). The *rgs1*Δ strain elaborates appressoria efficiently on both inductive and non-inductive surfaces [2], [29]. We asked if the specific expression of the DEP-DEP domain or the RGS domain in the *rgs1*Δ background could alter its response to surface cues.

**Figure 5:**
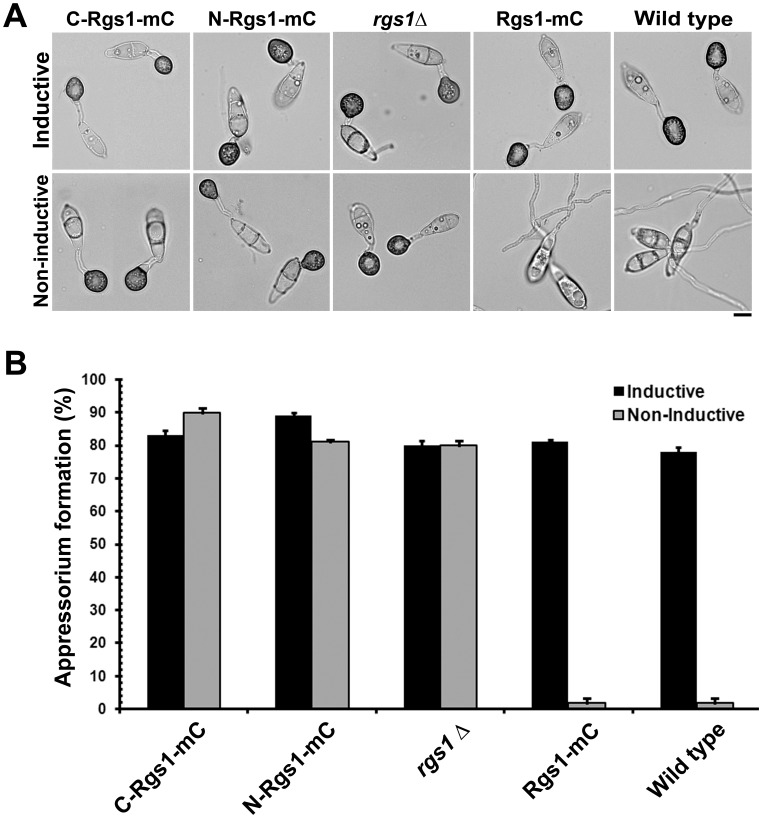
The domain expressing strains retain the ability to elaborate appressoria on non-inductive surfaces. Appressorium formation assays on inductive and non- inductive surfaces. (**A**) Conidia harvested from the C-Rgs1-mC, N-Rgs1-mC, *rgs1*Δ, Rgs1-mC and wild type were inoculated on inductive (plastic coverslips) and non-inductive (GelBond membrane) and evaluated for appressorium after 16 h. The C-Rgs1-mC, N-Rgs1-mC, and *rgs1*Δ strains also formed appressoria on non-inductive surfaces. Scale bar = 10 µm. (**B**) Bar graph illustrating the efficiency of appressorium formation in the C-Rgs1-mC, N-Rgs1-mC, *rgs1*Δ, Rgs1-mC or wild type on inductive (black bar) or non-inductive surfaces (gray bar). Data represents mean ± SE of three independent replicates.

We quantified the efficiency with which the aforementioned strains form appressoria on inductive and non-inductive surfaces. As expected ∼80% of wild type conidia and ∼80% of Rgs1-mC expressing conidia formed appressoria efficiently on inductive surfaces, and failed to do so on non-inductive surfaces (∼2% for both the strains) (Figure 5A and 5B). On the other hand, conidia from the C-Rgs1-mC expressing (DEP-DEP domain Δ) or the N-Rgs1-mC expressing (RGS domain Δ) strain formed appressoria with high efficiency on both inductive (83% and 89% respectively) as well as non-inductive surfaces (90% and 81% respectively), pheno-copying the *rgs1*Δ strain (80% on both surfaces). In conclusion, our results suggest that the expression of DEP or RGS domains individually fails to uncouple surface dependency from appressorium formation in the *rgs1*Δ.

### Infection Assays on Barley Explants and Rice Seedlings

**Figure 6:**
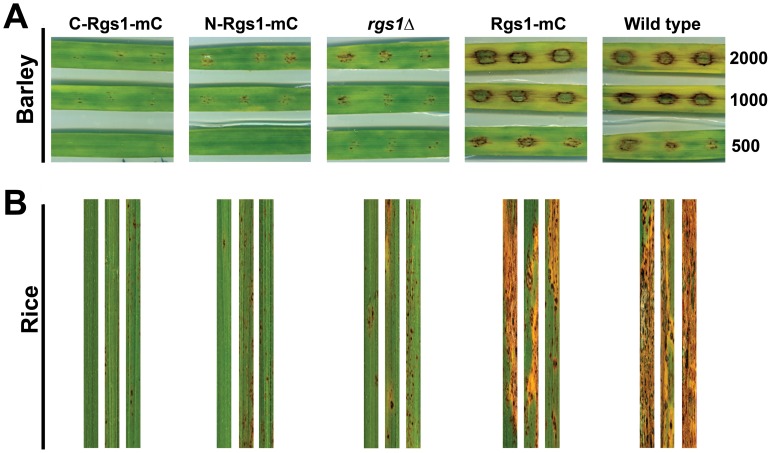
Pathogenicity related defects of *rgs1*Δ are not rescued by expression of independent domains fragments. Pathogenicity assays on barley leaf explants. (**A**) Barley leaf explants were inoculated in triplicate with specified number of conidia from the strains being tested. The numbers on the right indicate the number of conidia per inoculation site. The disease symptoms were assessed seven days post inoculation. (**B**) Two week old rice seedlings (cultivar CO39) were spray inoculated with conidia from the indicated strains, and the disease symptoms were evaluated after 9 days.

The *rgs1*Δ strain has been previously demonstrated to be defective in its ability to cause blast disease, and this defect has been attributed to the inability of the infection hyphae elaborated by the *rgs1*Δ to spread into the neighbouring cells within the host [28], [29]. In order to determine if either the DEP or the RGS domain is sufficient to rescue the *in planta* defects of the *rgs1*Δ, we inoculated barley leaf explants with conidia from wild type, Rgs1-mC, *rgs1*Δ, C-Rgs1and N-Rgs1 strains at various dilutions. The wild type and the *rgs1*Δ served as controls. The disease symptoms were evaluated seven days post inoculation (Figure 6A). We also evaluated the ability of the strains to cause disease in rice (variety CO39). For this assay, we spray inoculated three-week old rice seedlings with conidia from the strains being tested, and assessed the disease symptoms nine days post inoculation. Unlike the wild type and the Rgs1-mC strain, which formed characteristic spindle-shaped blast lesions with gray centres, the development of lesions as well as the progression of the disease was dramatically slow and not robust in both the domain deletion and *rgs1*Δ strain (Figure 6B). The reduced ability of the individual domain deletion strains or the *rgs1*Δ to cause disease on barley or rice was independent of the conidial load used. Taken together, the expression of the DEP or the RGS domain fails to rescue the pathogenicity related defects of the *rgs1*Δ strain.

### The DEP Domains Function to Target Rgs1 to Vesicular Compartments in *Magnaporthe*

**Figure 7:**
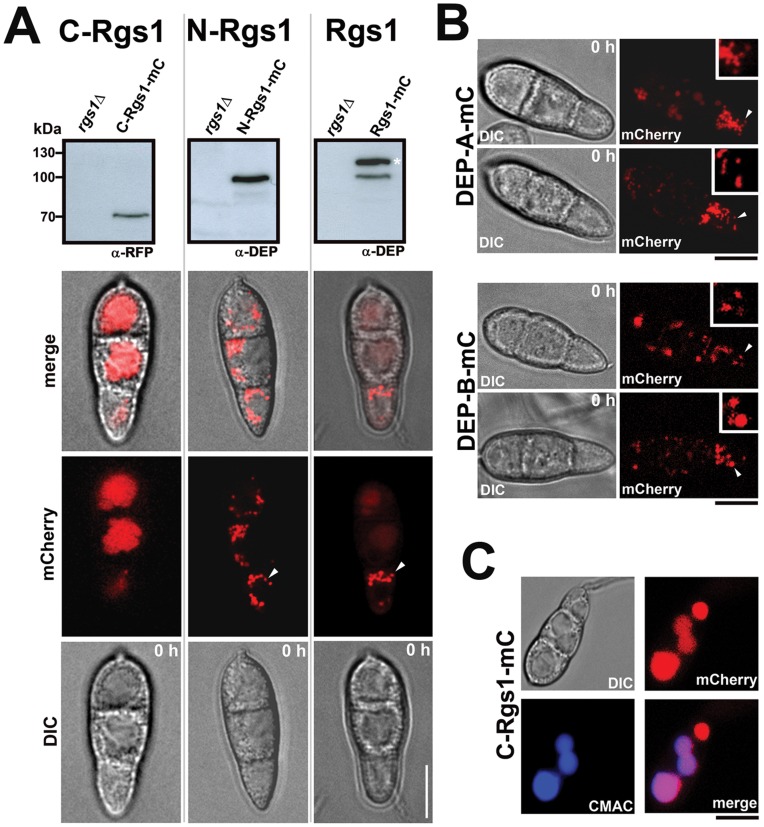
Specific targeting of Rgs1-mC to sub cellular vesicular structures is mediated by the N-terminal DEP-DEP domain. Subcellular localization and immuno-blot confirmation of protein expression. (**A**) Verification of expression of the mCherry tagged protein and individual domain fragments by western blot analysis and microscopy. Whole cell extracts were made from the *rgs1*Δ, Rgs1-mC, N-Rgs1-mC and C-Rgs1-mC strains cultured in CM. The protein samples were subjected to SDS-PAGE and analyzed by western blot using antisera against DEP-DEP domain (α-DEP) and mCherry (α-RFP). Specific immuno reactive bands corresponding to the full-length protein (Rgs1-mC), N-terminal DEP-DEP domain and C-terminal RGS domain were detected in each case. The *rgs1*Δ lysate served as a control. The white asterisk indicates a probable post-translationally modified form of Rgs1. Molecular mass standards (kDa) are indicted on the right. Intracellular localization of the individual domain fragments and the full-length Rgs1 protein. Conidia were harvested from the C-Rgs1-mC, N-Rgs1-mC and Rgs1-mC strains and microscopic observations were made at the indicated time points. DIC and wide field images were captured using the requisite filter sets. The arrowhead points to the punctate vesicles prevalent in the Rgs1-mC and N-Rgs1-mC strains at 0 h. Scale bar = 10 µm. (B) The DEP-A and DEP-B domain contribute to punctate/vesicular localization of Rgs1. Conidia from the indicated mCherry tagged strains were visualized using a wide field microscope and images captured at the indicated time point. The arrowheads in each image indicate the region of the conidium magnified and depicted as inset. Scale bar = 10 µm. (**C**) Vacuolar localization of the catalytic core domain (C-Rgs1-mC). Representative images from a colocalization experiment between the C-Rgs1-mC strain with a vacuole specific dye, CMAC. The merged panel suggests a predominant trafficking of the core RGS domain to the vacuole. Scale bar = 10 µm.

To aid visualization and to track the subcellular localization of the individual domains (DEP-DEP, DEP-A, DEP-B and RGS), the plasmid constructs expressing the domain fragments also incorporated a mCherry (mC) tag at the C-terminal end. Microscopic observations utilizing wide-field optics were made on freshly harvested conidia (Figure 7A, lower panel and 7B). In freshly harvested conidia (0 hpi), the full-length Rgs1-mC localized to distinct punctate vesicles in the terminal cell of the conidium. Interestingly, the N-Rgs1-mC (containing both the DEP domains), and the individual DEP domain fragments (DEP-A or DEP-B) localized to punctate vesicular structures in the conidia, identical to the full-length Rgs1-mC protein (Figure 7A and 7B). However, unlike the full-length Rgs1 protein that appeared to exclusively localize to the terminal cell of the conidium; the vesicular punctae in the strains expressing the N-Rgs1-mC, DEP-A-mC or DEP-B-mC, were evident throughout the conidium (Figure 7A and 7B). In addition, the DEP-B-mC containing punctae/vesicles displayed weaker signal intensity besides being fewer in number, compared to the DEP-A-mC containing vesicles (Figure 7B).

In sharp contrast, the C-terminal RGS catalytic core (C-Rgs1-mC) localized primarily to subcellular compartments which likely resembled vacuoles in the conidia (Figure 7A and 7C). To confirm that RGS domain indeed localized predominantly to vacuoles, co-localization experiments were carried out with the well-characterized vacuolar stain CMAC (7-amino-4-chloromethylcoumarin) [37]. As is evident from Figure 7C, Rgs1 protein lacking the N terminal DEP-DEP domain was primarily targeted to the vacuoles, unlike the intact full-length protein.

In addition to microscopically visualizing the tagged domains (C-Rgs1-mC and N-Rgs1-mC) and the full length protein (Rgs1-mC), we also confirmed their expression by western blot analysis. Using α-DEP antibody or α-RFP antisera (Figure 7A, upper panel), specific immuno reactive bands of expected molecular masses were detected in each case, Rgs1-mC (∼110 kDa), N-Rgs1-mC (∼105 kDa) and C-Rgs1-mC (∼70 kDa). Total protein lysates from the *rgs1*Δ strain acted as a control in all the cases.

Thus, we conclude that the N terminal DEP-DEP domains of *Magnaporthe* Rgs1 cooperatively play a critical role in facilitating the specific targeting of the core catalytic domain to specific vesicular compartments. In its absence, or upon endoproteolytic cleavage, the separated catalytic RGS domain is likely rendered inactive or non-functional, and trafficked to the vacuole possibly for degradation.

## Discussion

The cAMP mediated G protein signaling cascade has been among the most well studied and well-characterized pathways in the blast fungus *M. oryzae*. A number of genetic studies carried out over the last decade in *Magnaporthe* have implicated a number of proteins involved in cAMP signaling cascade and in regulating various aspects of asexual and pathogenic development [2], [3], [4], [38], [39], [40].

RGS proteins are essentially GTPase-activating proteins and represent a vital component of the G protein signaling cascade. They aid in accelerating the hydrolysis of GTP to GDP on the active Gα subunits and thus indirectly function to regulate the intensity and robustness of the signaling. Given that RGS proteins play a very important role in controlling G-protein signaling, the mechanisms by which RGS proteins themselves are regulated in filamentous fungi has not been addressed and is poorly understood.

The yeast RGS protein, Sst2, has been shown to be regulated by transcription and phosphorylation [41]. An additional post translational mechanism involving endoproteolytic cleavage has been established to regulate Sst2 function *in vivo* [34]. Here we demonstrate that *Magnaporthe* Rgs1 also undergoes endoproteolytic cleavage, in a manner similar to yeast Sst2. We further demonstrate that upon processing, *Magnaporthe* Rgs1 yields an N-terminal DEP domain that primarily functions to target the Gα-GTP interacting C-terminal RGS core domain to punctate vesicular structures. In the absence of the DEP-DEP domain the RGS domain is trafficked to the vacuole.

In order to determine the extent of conservation of the functional domains and putative endoproteolytic cleavage sites between filamentous fungi and yeast, we carried out comparative sequence alignment of full-length RGS proteins between the two species. We used Rgs1 from *M. oryzae*, FlbA from *A. nidulans* (representing the prototypical RGS from filamentous fungi), Sst2, from *S. cerevisiae*, and the fission yeast Rgs1. As previously described in literature [20], [30], all the four proteins have conserved N-terminal DEP domains and a C-terminal catalytic RGS domain (Figure 1A). The serine at position 415 and asparagine at 417 have been previously described as key residues where endoproteolytic cleavage likely takes place in Sst2 (Figure 1A and 1B, box and asterisks). Based on our alignments, we found the asparagine to be highly conserved in Sst2 orthologs in filamentous fungi; the serine however is replaced/substituted by aspartic acid. This substitution likely does not affect the efficiency of endoproteolytic processing of *Magnaporthe* Rgs1. Although we have not directly tested the effect(s) of point mutations in the putative cleavage site for *Magnaporthe* Rgs1, similar experiments carried out in yeast suggests that cleavage-site mutant Sst2p (in the implicated Ser and Asn) undergoes proteolytic processing in a manner identical to its wild-type Sst2 counterpart [34], suggestive of a more complex recognition mechanism and probable involvement of additional amino acids within or in the proximity of the predicted endoproteolytic cleavage site.

Given that the endoproteolytic cleavage site is likely conserved in *Magnaporthe* Rgs1, we carried out detailed immuno blot analysis with various *Magnaporthe* strains (wild type, Rgs1-OE and Rgs1-GFP) and antibodies to confirm Rgs1 processing and the existence of cleaved N and C terminal fragments. We detected cleaved N-terminal DEP and C-terminal RGS domain fragments for Rgs1. Taken together our data strongly suggests that, *Magnaporthe* utilizes endoproteolytic cleavage as a means to regulate Rgs1 function post translationally. Such a mechanism of functional regulation may be conserved across many other fungal species, although it remains to be experimentally verified. During our immuno-blot analysis (Figure 2A and 7A, marked with black and white asterisk respectively), we noticed a high molecular weight band (nearly ∼120 kDa) well above the expected molecular mass of the full length Rgs1 protein. To confirm the identity of the high molecular weight species, we carried out mass spectrometric analysis on the protein eluted from silver stained gels, and interestingly found that the protein indeed represented *Magnaporthe* Rgs1 protein (Figure S1, asterisk). Although not experimentally verified, we think that this species may represent a post translationally modified form of the full-length protein. This distinct species is likely masked in the Rgs1-over expression strain and poorly visible in the wild type (Figure 2A).

We next focused our experiments on understanding the functional significance of Rgs1 endoproteolytic cleavage. In order to do so, we expressed individual domain fragments (N and C terminal) in the *rgs1*Δ background. We further characterized the *rgs1*Δ as well as the individual domain deletion strains in order to address if the expression of either fragments in the mutant background would rescue the functionality of the full-length protein. We found that the domain deletion strains phenocopied all the defects exhibited by the *rgs1*Δ mutant in the assays tested such as colony morphology and aerial hyphal growth (Figure 3B), wettability of the colony surface (Figure 3C), hyper conidiation defects (Figure 4) appressorium formation and pathogenicity related defects (Figure 5 and 6). A possible explanation for the lack of rescue of the *rgs1*Δ phenotype by the individual domains is that the N and C terminal domains carry out distinct as well as likely independent functions. A similar observation has been made for the yeast Sst2, where in, expression of either N- terminal Sst2 or C-terminal Sst2 in the *sst2*Δ background, failed to rescue *SST2* function *in vivo* [34]. Thus, it is possible that endoproteolytic cleavage and vacuolar sequestration of the catalytic domain is a likely means of down regulating Rgs1 function in *Magnaporthe*.

Although we have not directly tested the above possibility, experimental evidence strongly suggests that co-expression of the independent domain fragments complements the *sst2*Δ phenotype only weakly. It has been further reasoned that this is likely due to limited ability of the membrane targeting DEP domain to contribute to the RGS core function *in trans* [42].

In order to verify the hypothesis that DEP-DEP and RGS domains carry out functionally distinct roles, we followed the subcellular localization of the individual domain fragments by wide-field microscopy. In the conidia, the double DEP (DEP-A and DEP-B in tandem) as well as individual DEP-A domain localized predominantly to punctate intracellular membranous structures, further substantiating a conserved membrane targeting function for the N-terminal domains. However, the comparatively weaker vesicular targeting (as evident by the diffuse signal of vesicles) ability of the DEP-B domain with respect to DEP-A, likely suggests a more important role for the non-canonical N-terminal DEP-A domain in specific vesicular targeting of Rgs1 in *Magnaporthe*. The full-length Rgs1, similar to the tagged DEP-DEP domain, predominantly localized to punctate intracellular structures. Faint vacuolar localization was also evident, which likely represented the proteolytically processed C-terminal portion of Rgs1 (Figure 7A). The intrinsic ability of the DEP domains for membrane targeting has been previously demonstrated. For example, the N-terminal portion of yeast Sst2 protein has been shown to be largely localized to microsomal membrane fractions [34]. Furthermore, Ballon *et al.,* in addition to substantiating the membrane targeting function for the DEP domain, have also demonstrated that the DEP domain aids the binding of Sst2 to the C-terminal tail of its cognate GPCR (Ste2) [42]. In mice, the DEP domain has been implicated in specifically targeting RGS9 protein to the outer segments of the rod photoreceptors [43]. Taken together, observations made in different model systems, suggests an important and conserved role for membrane targeting in the overall function of Rgs1.

The C-terminal RGS domain is crucial for the catalytic activity of the RGS protein. The RGS domain has been shown to be necessary and sufficient for the GAP activity of several Rgs proteins. When we looked at the localization of only the C-terminal RGS core domain in *Magnaporthe*, we found that it was mostly targeted to the vacuole as corroborated by co-staining with vacuole specific dye CMAC (Figure 7C). We hypothesize that the vacuolar targeting of the RGS domain is a likely consequence of its inability to be targeted to specific sites of action (membranous structures and the plasma membrane), due to the absence of a functional targeting DEP domain. It is also possible that the vacuolar targeting/sequestration facilitate the down regulation of Rgs1 catalytic activity.

In summary, we show that *Magnaporthe* Rgs1 undergoes limited proteolysis to yield an N-terminal DEP domain and a C-terminal catalytic core. The proteolytic cleavage may represent a conserved but not fully understood mechanism of post-translational modification (in addition to others) employed by filamentous fungi and yeast to regulate RGS protein function and G protein signaling. We further demonstrate a conserved and inherent membrane/vesicular structure targeting function for the N-terminal DEP-DEP domain of *Magnaporthe* Rgs1. Our findings also support a previous study that demonstrates functionally distinct although interdependent roles for the DEP-DEP and RGS domains [34]. Future studies would focus on identifying and characterizing the vesicular compartments to which Rgs1 and the cleaved DEP fragments localize.

## Materials and Methods

### Fungal Strains and Culture Conditions

The *M. oryzae* wild-type strain B157 was obtained from the Directorate of Rice Research (Hyderabad, India). Rgs1 deletion (*rgs1*Δ) strain was created by Liu Hao *et al*., as described [2]. For culture maintenance and conidiation, wild type and mutant strains were grown on prune agar medium (PA; per L: 40 mL prune juice, 5 g lactose, 5 g sucrose, 1 g yeast extract and 20 g agar, pH 6.5) as described [4]. Mycelial plugs were subcultured onto PA plates and incubated in a 28°C incubator in the dark for 7 days. For conidiation, cultures was incubated at 28°C in the dark for two days followed by light induction by exposing the plates to continuous fluorescent light at room temperature for 7 days.

### Surface Hydrophobicity Assays

To test the surface hydrophobicity of the fungal colonies, the strains being tested were grown on PA plates for 7 days at 28°C in the dark. Three 20 µl droplets of sterile distilled water were placed on the colony surface, moving from the centre towards the periphery. The colonies with the water droplets were subsequently incubated at room temperature.

### Conidiation Assay

Conidia were harvested by adding sterile distilled water to the light-exposed plates and scraping the surface of colonies with an inoculating loop. The suspension was filtered through two layers of Miracloth (Calbiochem, San Diego, USA), collected in Falcon tubes (BD Biosciences, USA). The spore suspension was vortexed for a minute to ensure complete detachment of conidia from the mycelia, and centrifuged for 10 minutes at 3,000 rpm at room temperature. The conidia pellet was resuspend in a fixed volume of distilled water. The radius of the colony was initially measured to calculate the surface area of the colony. Conidia produced by a given colony were quantified using a hemocytometer and reported as the total number of conidia present per unit area of the colony.

### Assessment of Appressorium Formation and Morphogenesis

For appressorium formation and morphogenesis assays, conidia were harvested and re-suspended at a concentration of 10^5^ conidia per mL in sterile water. 20 µl droplets of the conidial suspension were inoculated on plastic cover slips or hydrophilic side of GelBond membrane (Lonza Walkersville Inc., USA) and incubating in a humid chamber at room temperature. The total number of appressoria was quantified after 16 hpi (hours post inoculation). Microscopic observations were made using an Olympus BX51 wide field microscope.

### Evaluation of Pathogenicity

For pathogenicity assays, leaves from two week old barley seedlings were cut into smaller pieces (2–3 cm long) and washed in sterile water, following which the leaf bits were rinsed for 45 seconds in 40% ethanol. The leaf pieces were then washed twice with sterile antibiotic containing distilled water. The washed leaves were finally dried and placed on kinetin agar plates (2 mg/mL kinetin, 1% agar). Conidia were quantified and a dilution series of the conidial suspension was inoculated on detached barley leaves at the required concentrations. The samples were incubated in a humidified growth chamber with a 16 h light/8 h dark cycle at 22°C. Disease symptoms were assessed 5–7 days post inoculation.

### Nucleic Acid and Protein Related Methods

Standard molecular biology techniques were carried out as described [44]. Genomic DNA was extracted from fungal mycelium grown in liquid CM, using the Master Pure DNA purification kit (Epicenter Biotechnologies). Plasmid DNA was isolated from overnight grown bacterial cultures, using the Geneaid High Speed Plasmid Mini Kit. PCR amplifications were carried out using standard technique and appropriate primer pairs (Table 1). DNA and protein sequence homology searches were executed using the BLAST program [45] and sequence alignments were performed using Clustal W software (www.ebi.ac.uk/Tools/msa/clustalw2/) [46] and shading of multiple aligned files was done using Boxshade 3.2 (www.ch.embnet.org/software/BOX_doc.html). Protein domains were identified by SMART (http://smart.embl-heidelberg.de/).

**Table 1:** Sequences of oligonucleotide primers used for cloning purposes.

Gene amplified	Oligonucleotide Sequence of primers (5′ –3′)	Enzyme site
P27 promoter	CAGAGAgaattcATAAATGTAGGTATTACCTG (F)	*EcoR*I
P27 promoter	CAGAGAgaattcTTTGAAGATTGGGTTCCTAC (R)	*EcoR*I
P27 promoter	CAGAGAgaattcATAAATGTAGGTATTACCTG (F)	*EcoR*I
P27 promoter	CAGAGAcatatgTTTGAAGATTGGGTTCCTAC (R)	*Nde*I
P27 and *RGS1*	CAGAGAgagctc CGTAGCGAACAAATCC (R)	*Sac*I
*RGS1*	CAGAGAgaattcATGGACGACACCTCCCGCC (F)	*EcoR*I
*RGS1*	CAGAGAcccgggTAACCGTTGCGAGCGGCTT (R)	*Sma*I
mCherry	CAGAGAcccgggATGGTGAGCAAGGGCGAGG (F)	*Sma*I
mCherry	CAGAGAggatccTTACTTGTACAGCTCGTCCATGCCG (R)	*BamH*I
*BAR*	CAGAGAgagctcAGCCCAGAACGACGCCCG (F)	*Sac*I
*BAR*	CAGAGAcccgggGATCTCGGTGACGGGCAG (R)	*Sma*I
TrpC terminator	CAGAGAggatccACTTAACGTTACTGAAATCATCAA (F)	*BamH*I
TrpC terminator	CAGAGAtctagaCGAGCCCTCTAAACAAGTGT (R)	*Xba*I
*DEP-A*	CAGAGAgagctcGTTACGTGAAGCAAAAG (R)	*Sac*I
*DEP-B*	CAGAGAgagctcGACTCTTTTGCTTCACG (F)	*Sac*I

Restriction endonucleases sites introduced for cloning purposes are highlighted in lower case in each primer.

### Construction of Plasmid Vector for DEP-DEP Domain-mCherry

To understand the function of only the C-terminal catalytic domain (amino acids 532–714) of Rgs1 in *Magnaporthe*, plasmid constructs were made that specifically expressed only the N-terminal DEP-DEP domain of Rgs1 in fusion with Bar (Bialaphos resistance encoding protein) and the mCherry fluorescent protein. The construct was driven by the widely used P27 promoter [47], [48]. The portion of the gene encoding the DEP-DEP domain of Rgs1 (MGG_14517) fused to the BAR gene was produced by gene synthesis (GenScript, USA) and the fragment was flanked by the restriction enzymes *Nde*I and *Sma*I at the 5′ and 3′ ends respectively to aid in subsequent cloning procedures. The TrpC terminator was first cloned into the pFGL44 vector (*BamH*I/*Xba*I sites), and then the mCherry was inserted at the *Sma*I/*BamH*I sites of pFGL44-TrpC terminator to yield pFGL44-mCherry-TrpC terminator. Finally, the promoter and *DEP-DEP* domain-*BAR* fragment were inserted at the *EcoR*I/*Sma*I sites of pFGL44-mCherry-TrpC terminator. The sequence of the plasmid pFGL-*DEP-DEP* domain- *BAR-*mCherry-TrpC terminator was confirmed by sequencing and subsequently introduced into the *rgs1*Δ strain via *Agrobacterium* T-DNA-mediated transformation. Fungal transformants was selected based on resistance towards ammonium glufosinate (BM containing 40 µg/ml ammonium glufosinate, Cluzeau Info Labo, France). Transformants were visually screened for DEP-DEP domain-Bar*-* mCherry expression and confirmed by genomic DNA sequencing and western blot analysis.

### Generation of Plasmid Vector for RGS Domain-mCherry

To characterize the function of the N-terminal DEP-DEP domain (1–486 amino acids) of Rgs1 in *Magnaporthe*, an approach similar to the one mentioned above was adopted. Constructs were generated in which only the C-terminal RGS catalytic domain of Rgs1 in frame with Bar (Bialaphos resistance encoding protein) and the mCherry fluorescent protein was specifically expressed. The gene encoding the RGS domain fused to the BAR gene was generated by gene synthesis (GenScript, USA), and was flanked by *Nde*I and *Sma*I at the 5′ and 3′ ends respectively.

The promoter and fragment encoding the fusion of the RGS domain-BAR were inserted at the *EcoR*I/*Sma*I sites of into pFGL44-mCherry-TrpC terminator using a three way ligation approach to yield pFGL-*RGS* domain-*BAR-*mCherry. The final clones were confirmed by restriction enzyme digestion using appropriate restriction enzymes, followed by sequence analysis. The sequence confirmed clone was then introduced into the *rgs1*Δ strain via *Agrobacterium* T-DNA-mediated transformation. Transformants was selected based on resistance towards ammonium glufosinate (BM containing 40 µg/ml ammonium glufosinate, Cluzeau Info Labo, France). Fungal transformants were visually screened for Rgs1domain-Bar-mCherry expression and confirmed by sequencing of the genomic DNA.

### Plasmid Vector Generation for DEP-A-mCherry and DEP-B-mCherry Constructs

To further dissect the function of the DEP-A (aa 225–325) and DEP-B (aa 408–486) domains present in the DEP-DEP region of the Rgs1, plasmid constructs were generated by independently cloning and C-terminally tagging the individual domain fragments (DEP-A and DEP-B). The N-terminal portion of Rgs1 encoding amino acids 1–225 was retained in both the constructs. Briefly, the fragment encoding the P27 promoter and the DEP-A domain and the BAR encoding fragment was PCR amplified from the plasmid pFGL-*DEP-DEP* domain- *BAR-*mCherry-TrpC (described above) using appropriate primer pairs. Using a three-way ligation approach, the PCR fragments containing the promoter and the DEP-A domain (*EcoR*I-*Sac*I) was cloned into pFGL44-mCherry-TrpC terminator, along with BAR fragment (*Sac*I-*Sma*I) to get the final construct pFGL-*DEP-A* domain-*BAR-*mCherry-TrpC. A similar approach was taken to tag the DEP-B domain with mCherry at the C-terminus. Essentially, the fragment encoding the promoter and the N-terminal (1–225aa) region of Rgs1 was PCR amplified from pFGL-*DEP-DEP* domain- *BAR-*mCherry-TrpC and digested using appropriate primers and restriction enzymes (*EcoR*I-*Sac*I) respectively. The fragment encoding the DEP-B domain and the BAR fragments were PCR amplified from the same template plasmid and digested using the restriction enzymes *Sac*I and *Sma*I. Both the digested fragments were cloned into pFGL44-mCherry-TrpC terminator to yield pFGL-*DEP-B* domain- *BAR-*mCherry-TrpC. Both the final constructs were subjected to sequence analysis and upon confirmation, introduced into the *rgs1*Δ strain via *Agrobacterium* T-DNA-mediated transformation. Transformants was selected based on resistance towards ammonium glufosinate (Cluzeau Info Labo, France). Transformants were visually screened for DEP-A-Bar*-*mCherry and DEP-B-Bar*-*mCherry expression and further confirmed by genomic DNA sequencing.

### Plasmid Constructs for the Expression of RGS1–mCherry Fusion

The mCherry was PCR amplified from p-mCherry (Clontech, USA). The promoter and the RGS1 gene were amplified using PCR with genomic DNA extracted from *Magnaporthe* wild type B157 strain as template. The TrpC terminator was amplified using pFGL275 as template. The PCR products were digested with the appropriate restriction enzymes (New England Biolabs, Beverly, MA) and purified by gel elution using the Nucleospin Extract II kit (Machery-Nagel, Easton, PA). Using a three way ligation approach the mCherry (*Sma*I/*BamH*I ) fragment along with the TrpC terminator as a *BamH*I/*Xba*I fragment were cloned into the *Sma*I/*Xba*I sites of pFGL44 vector (encoding hygromycin phosphotransferase gene (*HPH1*)) to obtain pFGL44-mCherry-TrpC terminator. The digested and eluted *RGS1* (*EcoR*I/*Sma*I fragment) and promoter (*EcoR*I-*EcoR*I) fragments were sequentially cloned into pFGL44-mCherry-TrpC, to yield pFGL44-*RGS1*-mCherry-TrpC and subsequently to give pFGL44-*RGS1*-mCherry-TrpC, representing the final construct. The orientation of the promoter fragment in the final constructs was confirmed using *Kpn*I and *Bgl*II restriction enzymes. The final clones were subjected to sequence analysis and were then introduced into appropriate background strains via *Agrobacterium* T-DNA-mediated transformation. Fungal transformants was selected based on resistance towards hygromycin (CM containing 250 µg/ml hygromycin, A.G.Scientific Inc, USA). Fungal transformants were screened for Rgs1-mCherry expression under a fluorescence microscope and confirmed by the sequencing of the genomic DNA.

### Protein Extraction and Immuno Blot Analysis

Total protein extracts were obtained by grinding fungal mycelium (grown for three days in CM) in liquid nitrogen and re-suspending in 300 µl of extraction buffer (10 mM Na2HPO4 pH 7.0, 0.5% SDS, 1 mM DTT and 1 mM EDTA). Lysates were cleared by centrifugation at 12000 g for 20 min at 4°C. Protein concentrations in the supernatant were determined by the Bradford assay (Bio-Rad, USA). Protein sample from each extract was fractionated by SDS–PAGE, transferred onto a PVDF membrane (Millipore Corporation, USA) and immuno blotted with α-DEP antiserum (1∶1000 dilution), α-RFP (1∶1000) and α-GFP (1∶1000). Secondary antibodies conjugated to horseradish peroxidase were used at 1∶10000 dilutions. The Super Signal kit (Pierce, USA) was used to detect the chemi-luminescent signal as instructed.

### Light and Wide-field Microscopy

Brightfield as well fluorescence samples were observed using appropriate optics on the BX51 microscope (Olympus, Japan) equipped with a PlanAPO 100X/1.45 or UPlan FLN 60X/1.25 objective with appropriate filter sets. Images were captured using a Cool SNAP HQ camera (Photometrics, USA) and processed using MetaVue (Universal Imaging, USA) and Adobe Photoshop CS3 (Adobe Inc, USA).

## Supporting Information

Figure S1 **Silver-stained gel of proteins immuno-precipitated from the Rgs1-mC strain.** Whole cell extracts from the Rgs1-mC strain were subjected to immuno-precipitation with anti-RFP antibody, and resolved on an SDS-PAGE. Both the highlighted silver-stained bands (black arrows) represent Rgs1 protein, as confirmed by mass-spectrometric analysis. The lower band ∼100 kDa represents Rgs1-mC protein, while the band ∼120 kDa, highlighted by asterisk, and may represent a post-translationally modified form of Rgs1 protein.
(TIF)
