# Supplementary figures and images for: Structure-Function Analysis of Rgs1 in Magnaporthe oryzae: Role of DEP Domains in Subcellular Targeting
Source: PLoS One. 2012 Jul 19;7(7):e41084. doi: 10.1371/journal.pone.0041084 (PMC3426613; doi:10.1371/journal.pone.0041084)

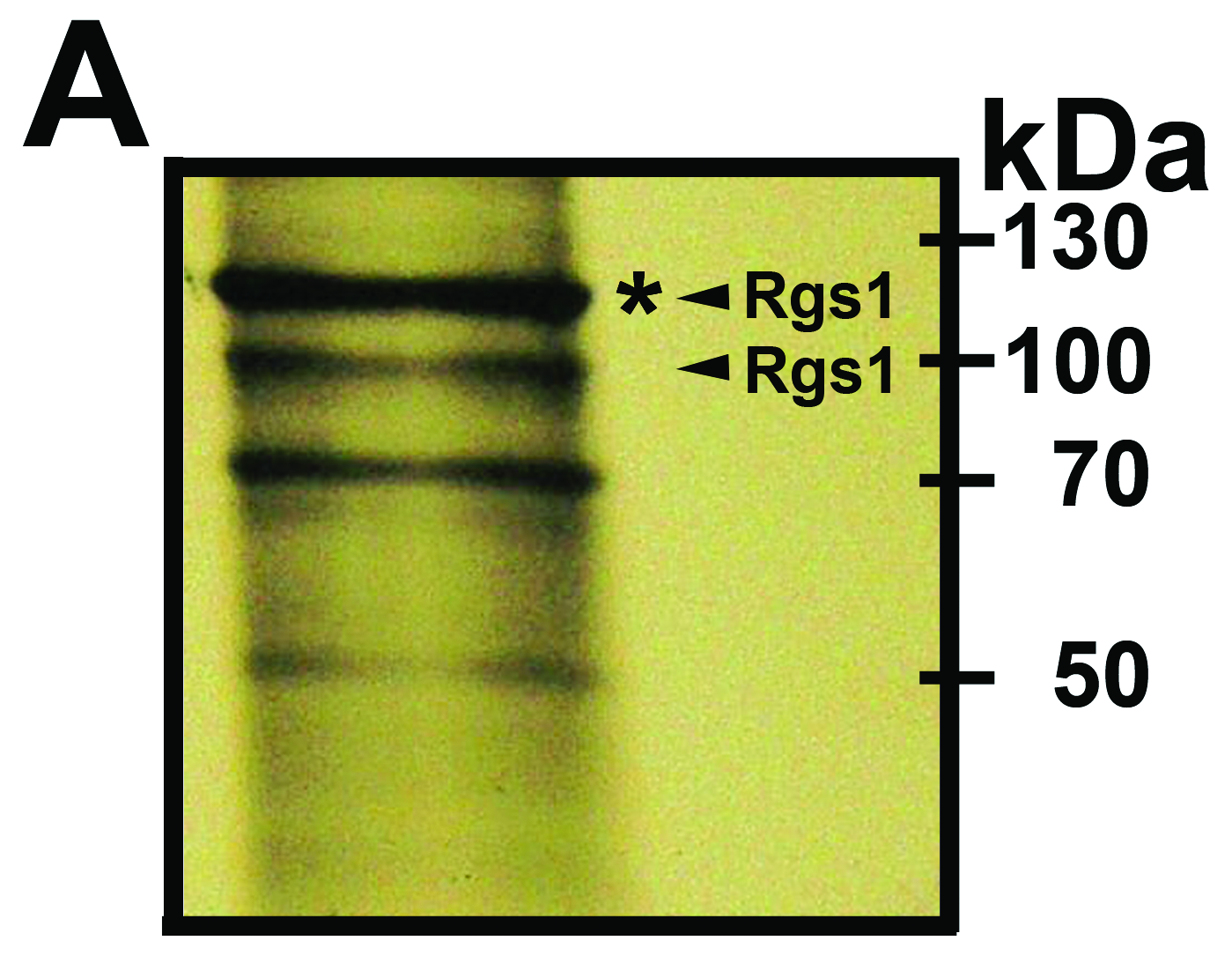

Supplement: Figure S1 — Silver-stained gel of proteins immuno-precipitated from the Rgs1-mC strain. Whole cell extracts from the Rgs1-mC strain were subjected to immuno-precipitation with anti-RFP antibody, and resolved on an SDS-PAGE. Both the highlighted silver-stained bands (black arrows) represent Rgs1 protein, as confirmed by mass-spectrometric analysis. The lower band ∼100 kDa represents Rgs1-mC protein, while the band ∼120 kDa, highlighted by asterisk, and may represent a post-translationally modified form of Rgs1 protein. (TIF) [file pone.0041084.s001.tif]
